# Supplementary material for: Implementation of CT Coronary Angiography as an Alternative to Invasive Coronary Angiography in the Diagnostic Work-Up of Non-Coronary Cardiac Surgery, Cardiomyopathy, Heart Failure and Ventricular Arrhythmias
Source: J Clin Med. 2021 May 28;10(11):2374. doi: 10.3390/jcm10112374 (PMC8199189; doi:10.3390/jcm10112374)
Supplement: Supplementary file 1 [file jcm-10-02374-s001.zip › jcm-1201661-supplementary.pdf]

Voorgeschreven IDR	Buisspanning (kVp)					
Gewicht	70 kVp	80 kVp	90 kVp	100 kVp	110 kVp	120 kVp
39 – 59 kg	1.01	1.20	1.39	1.60	1.80	2.00
60 – 74 kg	1.08	1.28	1.48	1.70	1.90	2.13
75 – 94 kg	1.27	1.50	1.74	2.00	2.24	2.50
95 – 109 kg	1.39	1.65	1.91	2.20	2.47	2.75

Schematic representation of the contrast delivery protocol. Iodine delivery rate (IDR) is given as in gram of iodine per second (g I/s) per body weight group and selected kVp. With the fixed injection speed of 6 ml/s and contrast material with 300 mg I/ml (Ultravist 300: iopromide 300 mg I/ml, Bayer Healthcare Pharmaceuticals, Whippany, USA), our maximum achievable IDR was 1.8 g I/s. The main bolus contrast volume for high-pitch spiral CTCA scans was 50 ml, and for prospective sequential step-and-shoot scans was 65 ml.
